# Nursing Staff Presenteeism Scale: Development and psychometric test

**DOI:** 10.1371/journal.pone.0301787

**Published:** 2024-04-16

**Authors:** Shiao-Pei Hung, Jin-Lain Ming, Mei-Yu Chang, Chi Wang, Chii Jeng

**Affiliations:** 1 School of Nursing, Taipei Medical University, Taipei, Taiwan; 2 Department of Nursing, Taipei Veterans General Hospital, Taipei, Taiwan; 3 School of Nursing, College of Nursing, National Yang Ming Chiao Tung University, Taipei, Taiwan; 4 Department of Nursing, Taichung Veterans General Hospital, Taichung, Taiwan; 5 Department of Nursing, Kaohsiung Veterans General Hospital, Kaohsiung, Taiwan; Universiti Putra Malaysia, MALAYSIA

## Abstract

**Background and objectives:**

Nurses tend to exhibit higher rates of presenteeism compared to other professions. Presenteeism can cause the work performance of nurses to suffer, jeopardizing their own and their patients’ safety and leading to decreased quality of care and increased risks of errors. However, there is a lack of a validated assessment tool for presenteeism in Taiwan. Thus, the purpose of this study was to develop a Nursing Staff Presenteeism Scale (NSPS).

**Methods:**

To develop questionnaire items, participants from three medical centers in Taiwan were recruited. Through convenience sampling, 500 nurses who met the selection criteria were recruited from November 1, 2022 to January 18, 2023. The scale was developed based on a systematic literature review, a previous study, and expert consultation, and 50 items were initially generated. After removing three items that lacked discriminative power, the reliability and validity of the remaining 47 items were evaluated. An exploratory factor analysis was used to establish the construct validity. A confirmatory factor analysis and structural equation modeling for cross-validation were used to assess relationships of factors with items and the overall NSPS.

**Results:**

The final scale consisted of 44 items assessed on a five-point Likert scale that loaded onto three different factors of physical or mental discomfort (18 items), work performance (15 items), and predisposing factors (11 items). These three factors were found to explain 63.14% of the cumulative variance. Cronbach’s alpha for the overall final scale was 0.953. The item-to-total correlation coefficients ranged 0.443 to 0.795.

**Conclusions:**

The NSPS exhibited satisfactory reliability and validity. It can be applied to assess the level of presenteeism among clinical nurses and provide medical institutions with information regarding the causes of presenteeism, predisposing factors, and the impacts of presenteeism on their work performance to enhance the safety and quality of clinical care.

## Introduction

The term presenteeism was proposed by Cooper [1]. It is related to the concept of absenteeism—specifically, it has the inverse meaning and refers to the phenomenon where a worker continues to attend work despite feeling unwell due to illness or fatigue caused by long working hours, leading to reduced productivity. The incidence of presenteeism varies from 30% to 90% across professions [2]. Nurses, compared to other professions, are more prone to this problem [3]. In China, 94.25% of nurses self-reported that they had been present but unwell within the past 6 months [4]. This might be attributable to an accountability culture in the healthcare industry [5].

Presenteeism is a global phenomenon and a major factor adversely affecting organizational performance [6]. Presenteeism has a major impact on nurses’ work ability and health status. It can also affect the quality of hospital services and the professional performance of medical teams and is closely associated with delayed recovery of patients [7]. When medical staff work while sick, they not only increase the risk of patient infections but also severely hinder patients’ recovery progress [5].

As health promoters, caregivers, and health knowledge advocates, nurses have high health literacy and should know that they should take sick leave and rest when feeling unwell. However, nurses are generally not familiar with the term presenteeism and are unaware of their own inappropriate behavior, becoming a typical representative of those who exhibit presenteeism [8]. Providing adequate support to nurses when unwell would reduce their stress and facilitate job crafting [9]. Effective supervisor support would also eliminate the stress caused by work-related issues [10] and further minimize the harmful consequences of presenteeism. Reducing nursing staff presenteeism is crucial to improve the patient experience and build a harmonious nurse-patient relationship [11].

Lin and Luo [12] reviewed and critiqued Western presenteeism research and identified three major flaws: confused definitions, a lack of measurement instruments, and a lack of a comprehensive theoretical framework. They recommended that future studies examine the cultural context to explore the impacts of presenteeism on employees in various workplaces and understand its implications for local management practices. One study conducted a comprehensive electronic database review and screened 1767 articles to identify appropriate measurement instruments for presenteeism. Eventually, three measurement instruments with the strongest level of evidence were selected, namely the Stanford Presenteeism Scale (SPS)-6, the Endicott Work Productivity Scale (EWPS), and the Health and Work Questionnaire (HWQ) [13]. However, these scales have notable limitations. For example, the SPS-6 only considers presenteeism related to physical discomfort but does not take mental distress into account. In addition, it is more applicable to nurses working in private institutions. Additionally, because of its limited number of items, it lacks sensitivity. The EWPS was designed to assess participants’ sensitivity to work productivity but does not specifically measure presenteeism. The HWQ primarily estimates participants’ life and work satisfaction over 1 week and also does not focus on measuring presenteeism.

The Nurse Presenteeism Questionnaire was developed by Shan et al. [14]. The scale focuses on presenteeism behavior but does not assess the impacts of presenteeism on work performance or productivity [15]. A systematic literature review and meta-analysis that examined 28 studies from 14 countries revealed that 50% of nurses showed up for work when unwell or unfit to work and proposed that effective measurement instruments for evaluating presenteeism among nurses and thoughtful solutions to presenteeism are needed [3]. To date, no gold standard measurement for presenteeism has been developed. The purposes of this study were to develop a Nursing Staff Presenteeism Scale (NSPS) and test its effectiveness.

## Methods

### Study design

A cross-sectional survey and descriptive research were conducted. The items for the NSPS were developed based on the theoretical framework for presenteeism proposed in our previous qualitative research and the relevant literature. Subsequently, psychometric tests and structural equation modeling (SEM)-based cross-validation were performed to establish the reliability and validity of the scale.

### Sample and setting

Participants were recruited by convenience sampling from three branches of Veterans General Hospital (medical centers) located in northern, central, and southern Taiwan. Recruitment was conducted in two stages. In the first stage, 200 nurses from a medical center in northern Taiwan were included. In the second stage, 300 nurses, 100 nurses from each of the three branches of Veterans General Hospital, were included (excluding those who had been recruited from the northern hospital branch in the first stage). The inclusion criteria were as follows: (1) being aged ≥ 20 years and (2) being a full-time employee. The exclusion criterion was having worked at the hospital for less than 6 months.

### Study procedures

**Fig 1:**
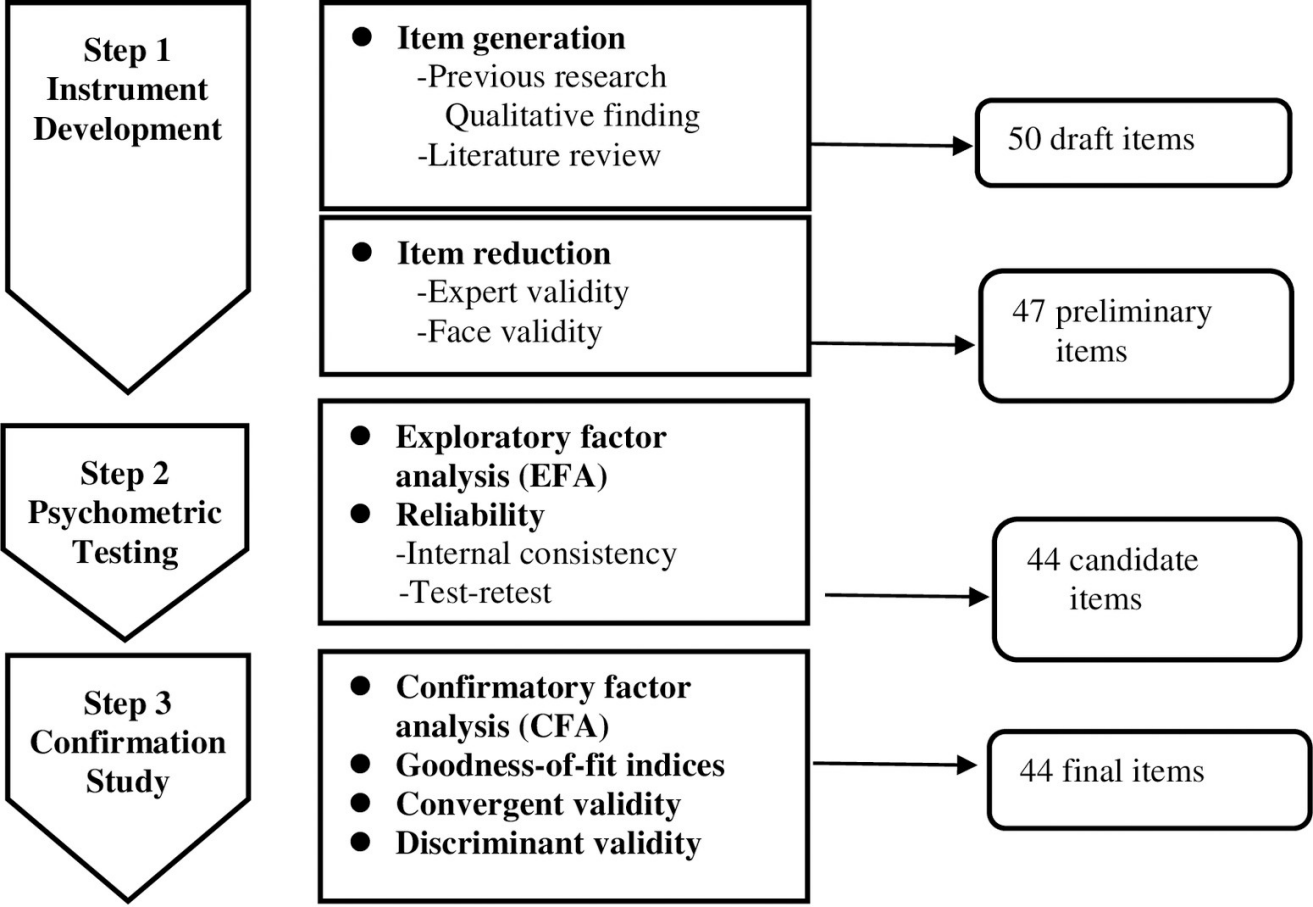
Development and validation of Nursing Staff Presenteeism Scale.

This study was conducted after approval from the Institutional Review Boards (IRBs) of the northern (no.: 2022-09-004C), central (no. CE22504B), and southern (no. KSVGH22-CT13-08) hospitals. Recruitment ran from November 1, 2022 to January 18, 2023. We contacted supervisors of nursing departments by telephone and email to explain the background and motivation of the study and seek their willingness to participate. Posters were put up in the hospitals for recruitment. After participants were selected, they provided informed written consent to participate in the research and data were collected. The research process was divided into three steps (Fig 1).

#### Step 1: Instrument development—Item generation and reduction

After referencing the qualitative results obtained in the study “The burden of being forced to work: a qualitative study of experience of presenteeism among nursing staff” and relevant literature obtained from the PubMed, Scopus, and CINAHL databases for the period 2000–2022, 50 draft items in total were selected for the NSPS.

To select the most important, applicable, representative, and clear scale items, five experts (scholars in the nursing field and clinical nurses with a master’s degree) were invited to conduct a two-round expert validity test on the draft items. Items with an item-level content validity index (I-CVI) of < .78 were removed from the scale [16]. An overall scale-level CVI of > .80 indicated that the scale had satisfactory content validity [17]. Then, face-to-face interviews with 20 participants were conducted for face validity testing, and items that they regarded as inappropriate were modified or removed. Draft items underwent expert validity and face validity testing, yielding the preliminary items.

#### Step 2: Psychometric testing

*Exploratory factor analysis (EFA)*. Preliminary items were scored on a five-point Likert scale, ranging from 1 (never) to 5 (always). A higher score indicated a higher prevalence of presenteeism. SPSS 22 (IBM, Armonk, NY, USA) was applied to analyze data of the preliminary items. Before the EFA, a Kaiser-Meyer-Olkin (KMO) test of sampling adequacy was performed (with a cutoff of > .50), and a significant result (*p* < .05) for Bartlett’s test was obtained. The suitability of the data for the EFA was thereby confirmed [18], and relationships between the items were assessed [19]. Factor extraction was performed. Factors with an eigenvalue of ≥ 1 were retained, and varimax orthogonal rotation was employed to obtain factors between the items. EFA and scree plot were repeatedly conducted. Item deletion and retention and the determination of the appropriate number of factors were based on criteria such as factor loadings (with a cutoff of ≥ .40) [20], the absence of cross-loading between items [18], and a minimum of three items for each factor [21]. The construct validity of the scale was thereby established.

*Reliability*. To assess the internal consistency of the scale, Cronbach’s α of > .70 was indicated to be acceptable [22]. A corrected item-to-total correlation coefficient of < .30 was used as a criterion for item deletion [23]. Further, 30 participants were selected, and their data were used to evaluate the test-retest reliability. The questionnaire was administered again to these participants 2 weeks after the initial test. The data were subjected to statistical analysis by calculating Spearman’s rank correlation coefficient (with a cutoff of ≥ .70; [23]), and a paired *t*-test (with a cutoff of *p* > .05) of the two sets of data performed to confirm the stability of the scale [24].

#### Step 3: Confirmation test

*Confirmatory factor analysis (CFA)*. To validate the EFA results, a CFA and SEM were used to establish relationships between the items and factors in the NSPS measurement model. AMOS 22 (IBM) was employed for the analysis, and the maximum likelihood estimation method was adopted. Before the analysis, the measurement model was assessed as to whether it was suitable for determining offending estimates for the SEM, while goodness-of-fit indices (GFIs), convergent validity, and discriminant validity were assessed to confirm the latent constructs between candidate items and the construct validity of the EFA results determined through cross-validation [18, 25]. In this way, the appropriateness of the measurement model was verified.

*Goodness-of-fit indices (GFIs)*. Validation of the model fit for the SEM does not have a consistent standard, and thus multiple fit indices should be considered [26]. In the present study, absolute fit induces were adopted: χ^2^ (*p >* .05) [27], χ^2^/df (with a cutoff of 1~5), and root mean square error of approximation (RMSEA; .05 < cutoff < .08, good fitting). Other fit indices were also included, namely the GFI, adjusted GFI (AGFI), comparative fit index (CFI), Tucker-Lewis index (TLI), and normed fit index (NFI). The cutoff value for model fit was ≥ .90 [28].

*Convergent validity*. When exploring the consistency between items for a latent variable and the correlations between latent variables (dimensions), the following three conditions for SEM convergence validity had to be met: (1) a standardized factor loading (λ) of ≥ .50 and a *t* value reaching significance (*p ≤* .05) [18]; (2) latent variables having a construct reliability of ≥ .70; and (3) latent variables having an average variance extracted (AVE) of ≥ .50 [29, 30].

*Discriminant validity*. The purpose of this analysis was to verify whether the latent variables belonged to a distinct category, thereby avoiding excessive overlap in their meanings [18]. The discriminant validity for the SEM had to meet the following criterion: the square root of the AVE of each latent variable should be greater than the correlation coefficient between the latent variables [30].

## Results

### Sample characteristics

We retrieved 500 valid questionnaires. The vast majority of participants were women (*N* = 477, 95.4%). Their ages ranged 22 to 61 years, with an average age of 37.0 years. Three-quarters held a college degree (77.0%). Their average job tenure was 13.9 years. Most of them worked on rotating shifts (69.0%). On average, they worked 4.9 days per week and 9.3 hours per day. Among the 500 cases, the data of 200 cases were subjected to an EFA statistical analysis, and the data of the remaining 300 cases were analyzed by a CFA. Distributions of case data of the two groups were roughly similar (Table 1).

**Table 1:** Demographic and nursing characteristics.

Item	Total (N = 500)		EFA(n = 200)	CFA(n = 300)
	Number (%)/M±SD		Number (%)/M±SD	Number (%)/M±SD
Gender				
Female	477(95.4%)		190(95.0%)	287(95.7%)
Male	23(4.6%)		10(5.0%)	13(4.3%)
Age (years)	37.0 ± 10.4		38.8 ± 10.0	35.8 ± 10.5
Education level				
Junior college	23(4.6%)		8(4.0%)	15(5.0%)
University/institute of technology	385(77.0%)		151(75.5%)	234(78.0%)
Graduate school	92(18.4%)		41(20.5%)	51(17.0%)
Job title				
Head nurse	40(8.0%)		17(8.5%)	23(7.7%)
Assistant head nurse	34(6.8%)		19(9.5%)	15(5.0%)
Registered nurse	426(85.2%)		164(82.0%)	262(87.3%)
Rank				
N0	40(8.0%)		12(6.0%)	28(9.3%)
N1	33(6.6%)		11(5.5%)	22(7.3%)
N2	224 (44.8%)		81(40.5%)	143(47.7%)
N3	81(16.2%)		34(17.0%)	47(15.7%)
N4	122 (24.4%)		62(31.0%)	60(20.0%)
Tenure (years)	13.9 ± 10.2		15.6 ± 10.1	12.7 ± 10.1
Work shifts				
Fixed shift	155(31.0%)		73(36.5%)	82(27.3%)
Rotating shift	345(69.0%)		127(63.5%)	218(72.7%)
Weekly working days	4.9 ± 0.6		4.8 ± 0.5	5.0 ± 0.6
Daily working hours	9.3 ± 1.2		9.51 ± 1.28	9.1 ± 1.1
Health status				
Very good	16(3.2%)		4(2.0%)	12(4.0%)
Good	109(21.8%)		33(16.5%)	76(25.3%)
Average	310(62.0%)		136(68.0%)	174(58.0%)
Poor	59(11.8%)		26(13.0%)	33(11.0%)
Very poor	6(1.2%)		1(0.5%)	5(1.7%)
Exercise habit				
None	236(47.2%)		95(47.5%)	141(47.0%)
1–3 time(s)/month	134(26.8%)		49(24.5%)	85(28.3%)
1–2 time(s)/week	97(19.4%)		40(20.0%)	57(19.0%)
3–4 times/week	26(5.2%)		12(6.0%)	14(4.7%)
Almost every day	7(1.4%)		4(2.0%)	3(1.0%)

M±SD: Mean ± standard deviation EFA: Exploratory Factor Analysis

CFA: Confirmatory Factor Analysis

#### Step 1: Instrument development—Item generation and reduction

According to a literature review and results of other qualitative research, we generated 50 draft items. After two rounds of expert validity testing, three items (i.e., B19, B20, and D15) with an I-CVI of < .78 were removed. The I-CVI and S-CVI of the remaining 47 items both reached 0.99. Subsequently, we conducted face validity testing with 20 participants, and they all deemed the wording and format of the items to be appropriate. The remaining 47 items were retained, yielding the preliminary items.

#### Step 2: Psychometric testing

*EFA results*. First, the 47 preliminary items were subjected to a KMO test for sampling adequacy and Bartlett’s test of sphericity. The KMO values were all ˃ .80, and Bartlett’s test results all reached significance (*p* < .001), indicating that the scale was suitable for a factor analysis. On the basis of results of four rounds of the EFA along with scree plot, three common factors were extracted. Three items (D10, D11, and D12) with factor loadings of < .40 were removed. No cross loading among factors was discerned. In this step, three items were removed, leaving a set of 44 candidate items. The eigenvalue of the 18 items for factor 1 was 14.755 and explained 38.83% of the variance. The eigenvalue of the 15 items for factor 2 was 5.821 and explained 15.31% of the variance. The eigenvalue of 11 items for factor 3 was 3.424 and explained 9.00% of the variance. The three factors explained 63.14% of the variance in total (Table 2).

**Table 2:** Explanatory factor analysis results (*N* = 200).

Item	Content	Physical or mental discomfort	Work performance	Predisposing factors
B07	I attend work as usual even if I feel dizzy or lightheaded.	.873		
B15	I attend work as usual even if I feel heavily stressed.	.870		
B13	I attend work as usual even if I feel emotionally down or experience feelings of depression.	.868		
B14	I attend work as usual even if I feel anxious or nervous.	.865		
B10	I attend work as usual even if I feel extremely tired.	.852		
B12	I attend work as usual even if I feel like I am getting sick.	.841		
B06	I attend work as usual even if I feel swelling, soreness, or heaviness in my legs.	.825		
B11	I attend work as usual even if I feel extremely weak.	.814		
B05	I attend work as usual even if I feel pain (e.g., headaches, menstrual pain, or stomachache).	.802		
B16	I attend work as usual even if I experience several days of insomnia or poor sleep.	.802		
B02	I attend work as usual even if I feel chest tightness or palpitations.	.801		
B04	I attend work as usual even if I feel muscle and bone soreness in my shoulders, back, or waist.	.796		
B03	I attend work as usual even if I experience diarrhea, constipation, or abdominal bloating.	.790		
B08	I attend work as usual even if I feel dizzy or lightheaded.	.778		
B01	I attend work as usual even if I experience coughing or symptoms of allergies or asthma.	.752		
B18	I attend work as usual even if I suffer an accident (e.g., minor car accident, sprain, or bruise).	.650		
B09	I attend work as usual even if I have a cold or fever.	.636		
B17	I attend work as usual even if I have not fully recovered after being discharged from the hospital.	.515		
C10	My responses become slow.		.824	
C09	I have difficulty concentrating.		.821	
C14	I have difficulty thinking clearly.		.803	
C08	I become easily distracted or absent-minded.		.802	
C12	I become easily irritated or angered.		.781	
C02	I interact less with my coworkers.		.763	
C11	I make more mistakes.		.756	
C04	I feel weak or lacking in energy.		.736	
C03	I become inpatient.		.734	
C01	I interact less with my patients.		.730	
C15	I become more prone to conflict with others.		.677	
C06	I need more time to complete my tasks.		.623	
C05	I want to leave work early.		.605	
C07	I simplify routine work processes on my own.		.523	
C13	I look forward to taking vacations more.		.463	
D04	My absence may be misunderstood as neglect of duties.			.773
D06	My absence may affect my performance evaluation.			.755
D01	My absence may burden my coworkers.			.736
D05	My absence may affect my salary/performance bonus.			.732
D02	My absence may affect the scheduled leave of others.			.732
D03	My absence may affect my scheduled leave.			.721
D07	If I were absent, no substitute personnel would be available to fill my position.			.702
D09	My absence may affect my relationships with coworkers.			.638
D08	If I were absent, no substitute personnel would be available to replace me and execute specific tasks.			.581
D14	Being present at work is part of professional ethics.			.482
D13	The reason for my absence may be questioned.			.424
	Eigenvalue	14.755	5.821	3.424
	Explained variance (%)	38.83	15.31	9.00
	Cumulative explained variance (%)	38.83	54.14	63.14

*Reliability*. Overall Cronbach’s α of the 44 candidate items was .953, and Cronbach’s α values for factors 1, 2, and 3 were .966, .936, and .886, respectively. The corrected item-to-total correlation coefficients of the 44 items ranged .443 to .795, all of which exceeded .30, indicating that the items were homogeneous with the overall concept of the scale. The internal consistency and reliability of the 44 items are displayed in Table 3. Spearman’s rank correlation coefficient of data obtained from 30 participants 2 weeks after the initial tests was .85. The test-retest results did not exhibit significant differences (*t* = 1.64, *p* = .172). This implied that the scale had high stability.

**Table 3:** Internal consistency for 44 items (*N* = 200).

Content		Cronbach’s α (if item were deleted)
**Factor 1. Physical or mental discomfort**		**.966**
B01	I attend work as usual even if I experience coughing or symptoms of allergies or asthma.	.965
B02	I attend work as usual even if I feel chest tightness or palpitations.	.964
B03	I attend work as usual even if I experience diarrhea, constipation, or abdominal bloating.	.964
B04	I attend work as usual even if I feel muscle and bone soreness in my shoulders, back, or waist.	.965
B05	I attend work as usual even if I feel pain (e.g., headaches, menstrual pain, or stomachache).	.964
B06	I attend work as usual even if I feel swelling, soreness, or heaviness in my legs.	.964
B07	I attend work as usual even if I feel dizzy or lightheaded.	.963
B08	I attend work as usual even if I feel dizzy or lightheaded.	.964
B09	I attend work as usual even if I have a cold or fever.	.966
B10	I attend work as usual even if I feel extremely tired.	.964
B11	I attend work as usual even if I feel extremely weak.	.964
B12	I attend work as usual even if I feel like I am getting sick.	.963
B13	I attend work as usual even if I feel emotionally down or experience feelings of depression.	.963
B14	I attend work as usual even if I feel anxious or nervous.	.963
B15	I attend work as usual even if I feel heavily stressed.	.963
B16	I attend work as usual even if I experience several days of insomnia or poor sleep.	.964
B17	I attend work as usual even if I have not fully recovered after being discharged from the hospital.	.968
B18	I attend work as usual even if I suffer an accident (e.g., minor car accident, sprain, or bruise).	.966
**Factor 2. Work performance**		**.936**
C01	I interact less with my patients.	.932
C02	I interact less with my coworkers.	.931
C03	I become inpatient.	.932
C04	I feel weak or lacking in energy.	.930
C05	I want to leave work early.	.934
C06	I need more time to complete my tasks.	.934
C07	I simplify routine work processes on my own.	.937
C08	I become easily distracted or absent-minded.	.929
C09	I have difficulty concentrating.	.929
C10	My responses become slow.	.929
C11	I make more mistakes.	.931
C12	I become easily irritated or angered.	.929
C13	I look forward to taking vacations more.	.938
C14	I have difficulty thinking clearly.	.929
C15	I become more prone to conflict with others.	.933
**Factor 3. Predisposing factors**		**.886**
D01	My absence may burden my coworkers.	.872
D02	My absence may affect the scheduled leave of others.	.873
D03	My absence may affect my scheduled leave.	.874
D04	My absence may be misunderstood as neglect of duties.	.867
D05	My absence may affect my salary/performance bonus.	.871
D06	My absence may affect my performance evaluation.	.870
D07	If I were absent, no substitute personnel would be available to fill my position.	.873
D08	If I were absent, no substitute personnel would be available to replace me and execute specific tasks.	.880
D09	My absence may affect my relationships with coworkers.	.875
D13	The reason for my absence may be questioned.	.885
D14	Being present at work is part of professional ethics.	.892
**Overall Cronbach’s α**		**.953**

#### Step 3: Confirmation testing

*CFA results*. To test the NSPS, the variance of the standard errors of all items ranged .183 to 1.656, all of which were > 0, and all *t* values reached significance (*p* < .05). In addition, the standardized regression weights (λ) of items ranged .610 to .948, with no abnormal values exceeding 1. The variance of error terms ranged .018 to .137, indicating that the NSPS measurement model did not have offending estimates and was suitable for fit index testing through SEM (Table 4).

**Table 4:** Confirmation study results (*N* = 300).

Latent variable	Item	λ	Error variable			Convergent validity			Discriminant validity	
			Variance	SE	*t* value	CR	AVE	λ^2^	√_AVE_	Correlation
Physical or mental discomfort						0.973	0.667			
	B01	0.761	0.873	0.073	11.964***			0.579		
	B02	0.730	1.063	0.088	12.013***			0.533		
	B03	0.764	0.820	0.069	11.959***			0.584		
	B04	0.823	0.459	0.039	11.814***			0.677		
	B05	0.840	0.478	0.041	11.753***			0.706		
	B06	0.863	0.402	0.035	11.643***			0.745		
	B07	0.827	0.690	0.058	11.803***			0.684		
	B08	0.835	0.725	0.062	11.772***			0.697		
	B09	0.706	1.110	0.092	12.066***			0.469	0.817	0.522^a^
	B10	0.900	0.298	0.026	11.368***			0.810		
	B11	0.892	0.436	0.038	11.447***			0.796		
	B12	0.902	0.357	0.031	11.349***			0.814		
	B13	0.934	0.262	0.024	10.829***			0.872		
	B14	0.946	0.196	0.019	10.475***			0.895		
	B15	0.948	0.183	0.018	10.416***			0.899		
	B16	0.903	0.365	0.032	11.338***			0.815		
	B17	0.610	1.656	0.137	12.126***			0.372		
	B18	0.781	1.078	0.130	12.143***			0.538		
Work performance						0.949	0.560			
	C01	0.711	0.480	0.041	11.783***			0.506		
	C02	0.727	0.443	0.038	11.739***			0.529		
	C03	0.758	0.410	0.035	11.633***			0.575		
	C04	0.747	0.523	0.045	11.673***			0.558		
	C05	0.616	0.756	0.063	11.971***			0.379		
	C06	0.721	0.525	0.045	11.756***			0.520		
	C07	0.741	0.745	0.062	12.085***			0.264		
	C08	0.900	0.201	0.020	10.294***			0.810	0.748	0.557^b^
	C09	0.912	0.190	0.019	9.986***			0.832		
	C10	0.891	0.233	0.022	10.493***			0.794		
	C11	0.784	0.332	0.029	11.525***			0.615		
	C12	0.724	0.508	0.043	11.749***			0.524		
	C13	0.755	0.782	0.065	12.054***			0.299		
	C14	0.855	0.318	0.029	11.008***			0.731		
	C15	0.705	0.550	0.046	11.887***			0.442		
Predisposing factors						0.920	0.539			
	D01	0.869	0.556	0.056	9.893***			0.755		
	D02	0.874	0.553	0.056	9.796***			0.764		
	D03	0.795	0.836	0.076	10.949***			0.632		
	D04	0.735	0.933	0.082	11.363***			0.540		
	D05	0.723	0.992	0.087	11.42***			0.523		
	D06	0.730	0.936	0.082	11.384***			0.533	0.734	0.544^c^
	D07	0.841	0.698	0.067	10.407***			0.707		
	D08	0.767	1.039	0.090	11.612***			0.457		
	D09	0.636	0.893	0.076	11.735***			0.404		
	D13	0.744	1.296	0.107	12.066***			0.195		
	D14	0.721	1.324	0.108	12.215***			0.042		

* *p* < .05

** *p* < .01

*** *p* < .001

λ, Standardized regression weights (factor loading).

AVE, average variance extracted.

CR, construct reliability.

SE, standard error.

√_AVE_, square root of AVE.

a, Physical or mental discomfort–work performance correlation coefficient.

b, Physical or mental discomfort–predisposing factors correlation coefficient.

c, Work performance–predisposing factors correlation coefficient.

*GFIs*. SEM was applied to examine the GFIs of the NSPS measurement model. χ^2^ test results revealed significant differences because of the excessively large sample size and thus, did not meet the criterion for goodness-of-fit. However, other indices revealed good fits: RMSEA = .076, which was below the criterion of .08; GFI = .859 and AGFI = .864, which were close to .90, the criterion of acceptable fit; and CFI, TLI, and NFI all exceeded .90, meeting the good-fit criterion. Additionally, SRMR = .071, which was below .08, indicating a good fit. The ratio of χ^2^ to degrees of freedom (χ^2^*/df*) was 4.12, which fell within the values of 1 to 5, implying a good fit. The results demonstrated that the measurement model had a reasonable fit (Table 5).

**Table 5:** Model goodness-of-fit.

Index	Cutoff	Evaluation	
		Value	Rank
Absolute Fit:			
χ^2^	*p*>.05	3706.419***	-
df	-	899	-
χ^2^/df	1–5	4.12	Good fit
GFI	≧.90	.859	Acceptable
AGFI	≧.90	.864	Acceptable
SRMR	≦.08	.071	Good fit
RMSEA	≦.08	.076	Good fit
Incremental Fit:			
NFI	≧.90	.945	Good fit
TLI	≧.90	.931	Good fit
CFI	≧.90	.944	Good fit

* *p* < .05

** *p* < .01

*** *p* < .001.

χ^2^, Chi-squared; df, degrees of freedom; GFI, goodness-of-fit index; AGFI, adjusted GFI; SRMR, standardized root mean square residual; RMSEA, root mean square error of approximation; NFI, normed-fit index; TLI, Tucker-Lewis index; CFI, comparative fix index.

*Convergent validity*. In the SEM, only three items had a standardized factor loading (λ) slightly below the cutoff of .70, namely B17 (λ = .610), C05 (λ = .616), and D09 (λ = .636). The standardized factor loadings of the remaining 41 items all met the criterion (i.e., a cutoff of ≥ .70). The construct reliabilities of the latent variables for factors 1, 2, and 3 were .973, .949, and .920, respectively, all of which met the threshold of ≥ 0.70. Further, the AVE of the latent variables was between .539 and .667, with all exceeding .50. This indicated satisfactory convergent validity between items and their respective latent variables (Table 4).

*Discriminant validity*. The square root of AVE values of the three latent variables (.817, .748, and .734) were all greater than the correlation coefficients between the latent variables (.522, .557, and .544). This implied that the items for various latent variables in the SEM model had satisfactory discriminant validity. The latent variables (constructs) were categorically distinct (Table 4).

**Fig 2:**
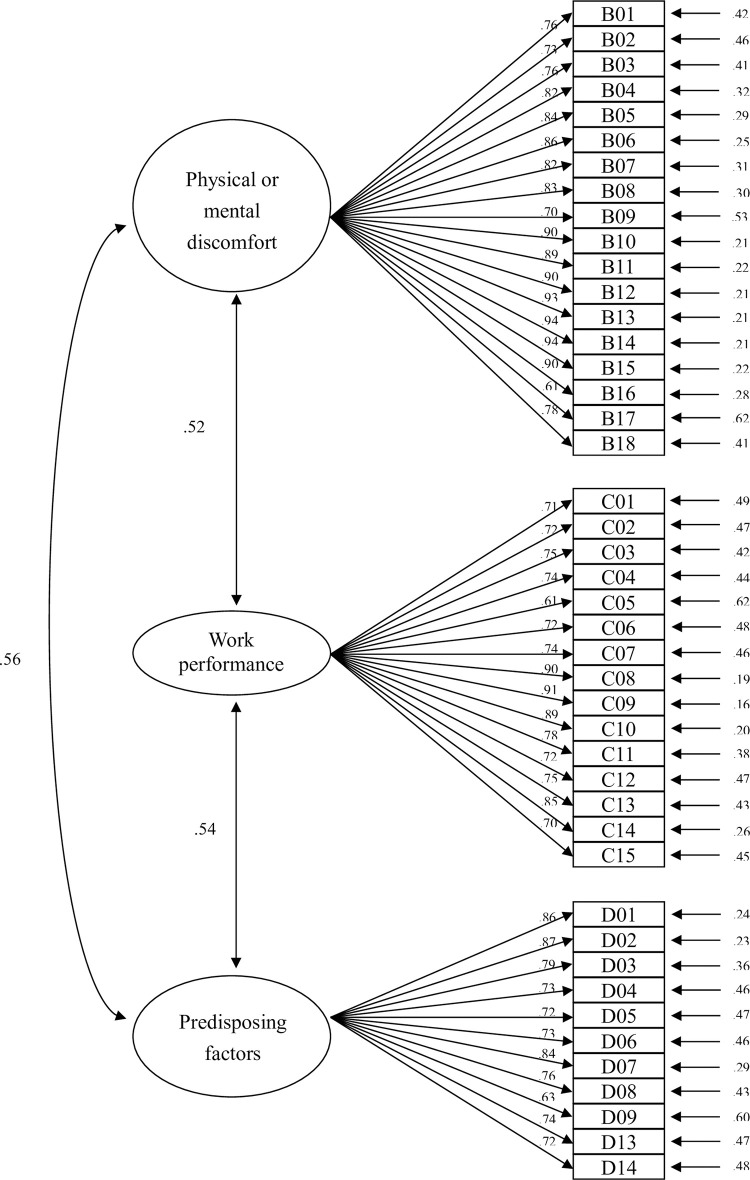
Nursing Staff Presenteeism Scale confirmatory factor analysis.

The NSPS measurement model had no offending estimates and demonstrated satisfactory GFIs, convergent validity, and discriminant validity. These results demonstrated that the measurement model possessed satisfactory internal and external quality for the SEM. Fig 2 displays CFA results of the NSPS measurement model.

## Discussion

This study developed a 44-item scale (NSPS) for assessing presenteeism among nurses. Three factors were generated using the EFA, each with high associated factor loadings, meeting the recommended value of 0.4 for all factor loadings [20]. In addition, the cumulative variation of the three factors was 63.14%, which was higher than the accepted standard of 40% [31]. High Cronbach’s alpha values for the overall scale and for the three factors indicated satisfactory reliability. A CFA was employed to validate results of the EFA, and the measurement model was constructed using SEM. Results indicated good model fit, convergent validity, and discriminant validity. Previous research pointed out that presenteeism lacks clear definitions, and a comprehensive theoretical framework [12], and existing assessment tools focus on presenteeism behavior but cannot assess the impact of presenteeism on work performance or productivity [15]. In this study, the NSPS is not only developed based on the theoretical framework constructed by previous qualitative research, but can also measure the state of work performance and the triggering factors of presenteeism.

Recently, a new presenteeism instrument containing 17 items and three dimensions (imperfect cognitive presence, imperfect emotional presence, and imperfect movement presence) with favorable validation characteristics was developed by Mohammadi et al. [32]. The instrument was able to explain 56.375% of the total variance. However, the contents of the three factors of this new tool are similar to factor 2: work performance in NSPS, while NSPS further covers the causes (factor 1) and triggering factors (factor 3) of presenteeism, allowing for a broader measurement of presenteeism. In addition, the NSPS has the following strengths: (1) Samples were collected from nursing staff at three medical centers located in northern, central, and southern Taiwan. Recruitment was conducted by putting up posters, and this avoided any hierarchical influence and respected participants’ autonomy. Nursing staff participated in this study voluntarily; and the sample was representative. (2) The development process of the NSPS was rigorous. Cross-validation was conducted using an EFA and CFA.

The final NSPS included three factors: physical or mental discomfort, work performance, and predisposing factors. These three factors represent the conditions, work efficiency, and motivating factors associated with presenteeism. The physical or mental discomfort factor had the highest explanatory power (38.83%), which compellingly suggests that nurses tend to work even when experiencing health symptoms or physical or mental discomfort in the belief that they can power through, leading to presenteeism. Rainbow et al. [33] determined that the causes of presenteeism among nurses are often mild early illness symptoms, including allergies, flu, headaches, lower back pain, asthma, stress, and depression. These conditions are often not taken seriously and immediately handled by nurses because they believe that these illnesses are not serious enough to affect their ability to work. More than 50% of nurses have reported experiencing poor physical and mental health conditions [34], and over 60% of nurses employed maladaptive coping mechanisms, such as consuming more junk food than usual, to relieve work-related stress [35]. In a qualitative study, respondents opined, “We constantly give patients advice, but in the end, we fail to apply it to ourselves” [8]. This is indicative of nurses’ neglect of or irrational attitudes toward their own health. One interesting study exploring the impact of menopausal symptoms on presenteeism found no significant association between physical and genitourinary symptoms and presenteeism after controlling for psychological symptoms. The findings indicate that menopausal symptoms, especially psychological symptoms, have a significant impact on presenteeism among menopausal women. Organizations need to address menopausal symptoms in the workplace, with a focus on reducing work-related stress among women with menopausal symptoms [36].

The work performance factor refers to problems that result from presenteeism, which affect job performance and interpersonal interactions. The associations between presenteeism and work performance are strong, even after multiple adjustments for other relevant variables [37]. Feelings of impatience, loss of enthusiasm for work, and increased likelihood of making mistakes may occur. A previous study reported that nurses who work while ill have significantly higher chances of making mistakes in the workplace compared to their healthy coworkers [33]. It was demonstrated that working while sick is associated with medication errors, patient falls, disease transmission, and iatrogenic infections [38]. We also noted that nurses often attempt to complete their tasks quickly when they force themselves to attend work despite feeling discomfort. However, this can backfire because they may end up overlooking critical aspects of their work and have to spend more time to compensate for deficiencies and complete tasks properly. In addition, their interaction time with patients may be shortened or limited when working in a weakened condition. Nurses become prone to giving brief and stock responses to patients’ inquiries without showing genuine empathy or carefully listening to their patients. These findings are consistent with a previous study which showed that when nurses are overworked, their basic care is inadequate in terms of medication management, health education, and nutritional intake [33].

Predisposing factors are related to nurses’ presenteeism behavior being affected by their team members and the overall work environment (e.g., attendance evaluation and ethics). For example, item D2, *My absence may affect the scheduled leave of others*, reflects that when a nurse takes sudden leave, the administrative supervisor has to recall staff who are currently on leave to fill the staffing gap. Item D5, *My absence may affect my salary/performance bonus*, refers to the situation where nurses are concerned that taking sudden leave might result in a deduction from their salary or performance bonus. Shan et al. [4] pointed out that in order to ensure ideal financial returns, nursing staff have a strong willingness to complete their work and refuse to take leave. Item D14, *Being present at work is part of professional ethics*, means that nursing staff believe that they can overcome physical and mental discomfort and be loyal to their personal work values. Regarding the tendency of nurses to attend work when ill, experts ascribe this to nurses’ notion of their work being a “calling” or “vocation” rather than a mere “job” [39]. Values of loyalty to peers and teamwork are prevalent among nurses, and this leads to the “super-nurse phenomenon” [40]. Laranjeira et al. [8] confirmed that nurses persist in working even when their physical or mental health is compromised; as a respondent opined, “We run ourselves to the very end, until you just can’t deal with it anymore.” This is consistent with the results of our previous study, which indicated that nurses exhibit a sense of responsibility to safeguard public health and thus remain dedicated to their work, even to the point of neglecting their own physical and mental well-being.

## Limitations

In this study, we only focused on nurses at three large-scale public medical centers. Further testing is required to determine the application of the NSPS to other private or smaller-scale hospitals to enhance the external validity and generalizability of the NSPS. In addition, recruitment was conducted by putting up posters in nursing stations. Potential participants might have been on leave and missed the information, leading to biases in the results. Additionally, this study did not include part-time employees, and the results must be verified for this group in the future.

## Conclusions

In this study, the NSPS was confirmed to be an instrument with satisfactory reliability and validity. The NSPS developed in this study can serve as an instrument for medical institutions to use to assess the level of presenteeism among nurses, as well as its causes, predisposing factors, and impacts on work performance. On the basis of those findings, institutions can establish effective improvement strategies. For example, with the use of the NSPS, medical institutions can learn the actual physical and mental conditions of nurses, implement preventive measures for chronic diseases, adjust nurse staffing accordingly, provide paid sick leave, and offer free psychological counseling. Such measures would reduce presenteeism among nurses and better ensure high-quality patient care. In the further research, receiver operating characteristics curves could be employed to determine an optimal cutoff score for the NSPS. This would facilitate the early identification of presenteeism among nurses and implementation of effective preventative measures.

## Supporting information

S1 Dataset (SAV)
